# Breaking the obesity-cancer link: assessing European school-based and complementary obesity-prevention strategies and their implementation obstacles

**DOI:** 10.3389/fpubh.2026.1791618

**Published:** 2026-09-10

**Authors:** Ioannis Rallis, Theodora Spyropoulou, Anastasios Doulamis, Aikaterini Angeli, George Kopsiaftis, Vassiliki Papaevangelou, Nikolaos Doulamis

**Affiliations:** 1 Spatial Intelligence Group, Institute of Communications and Computer Systems, National Technical University of Athens, Zografou-Athens, Greece; 2 Laboratory of Photogrammetry and Computer Vision, Department of Topography, School of Rural, Surveying and Geoinformatics Engineering, National Technical University of Athens, Zografou-Athens, Greece; 3 Third Department of Pediatrics, ATTIKON General University Hospital, National and Kapodistrian University of Athens, Haidari-Athens, Greece

**Keywords:** cancer risk prevention, childhood obesity, comparative European analysis, programme implementation, public health policy, scalability, school-based interventions

## Abstract

Nowadays, obesity among children and adolescents is considered an additional factor in cancer development during adulthood. These facts are supported by current epidemiological studies. To address these challenges, a wide range of obesity prevention programmes have been launched in Europe, promoting actions for personalized healthy dietary behaviors, physical exercise, and combinations of both, often using e-health platforms. Another pillar of interventions is school-based policies, such as limiting access to high-fat and high-sugar foods or providing healthy food vouchers, which represent another pillar of public health action, complemented by actions that cover out-of-school activities, such as family interventions, tax policies, engagement actions, and so on. However, the main limitations of the current intervention policies are their depth of penetration and, consequently, upscaling issues. Furthermore, there is a lack of a concrete approach to assess these policies within a Multi-Actor, Context-Aware framework. This multi-pillar assessment is presented in this paper for evaluating children's/adolescents' obesity programmes across fourteen European countries. The analysis includes the coverage of each programme, its financial limitations, results obtained in terms of obesity reduction and mitigation of cancer risk, along with structural and educational system limitations.

## 1 Introduction

Obesity is widely recognized as one of the most severe global epidemics, affecting nearly every country and showing rising prevalence despite extensive public health campaigns and interventions (1). According to the World Health Organization (WHO), the global prevalence of obesity has almost tripled since 1975, with projections indicating that

these trends will likely continue (2–4). Within this broader context, childhood obesity has emerged as a particularly acute public health challenge in Europe, with recent data confirming that approximately one in three children aged 6 to 9 is overweight or obese (5–7).

A robust body of epidemiological and clinical evidence supports the direct association between obesity—especially childhood obesity—and the development of various types of cancer in later life (8). Data from the Centers for Disease Control and Prevention indicate that excess body weight is linked to more than thirteen different cancers, including gastrointestinal, kidney, uterine, and ovarian malignancies. Longitudinal studies have further demonstrated that elevated body mass index (BMI) in adolescence is not only associated with increased cancer risk but is also predictive of greater morbidity and mortality from cancer during a 45-year follow-up period (9). Notably, an increased hazard ratio for cancer is evident above the 75th BMI percentile during adolescence, whereas cardiovascular risks become significant above the 50th percentile. This evidence is also verified by epidemiological studies (10). This emphasizes the urgency for effective, early-life interventions that can modify risk trajectories before irreversible health impacts ensue.

To address these challenges, a wide array of intervention programs and research projects have been launched across Europe and beyond, targeting obesity prevention among children and adolescents through multifaceted approaches. The majority of these programs are targeted at the individual level, promoting actions for personalized healthy dietary behaviors, physical exercise and combinations of them (11), usually exploiting e-health platforms (12). In addition, school-based policies, such as limiting access to high-fat and high-sugar foods or providing healthy food vouchers, represent another pillar of public health action (13). Furthermore, actions outside the school community have been investigated, mainly focusing on community interventions (community consultation and engagement processes with school, family, local health providers, sport activities organization, and government health) (14). However, the main limitations of the current intervention policies for controlling obesity in childhood and adolescents is implementation effectiveness, the penetration depth of the policies and consequently upscaling issues.

This paper envisions to address the current barriers and obstacles toward an effective implementation of the above-mentioned interventions for controlling obesity in childhood and adolescence by (i) identifying current intervention policies and their impact in obesity reduction, (ii) assessing these policies under a *Multi-Actor, Context-Aware framework* and (iii) finally proposing holistic measures and actions that can be useful in obesity reduction and cancer control management for the adulthood. The paper is focused on policies and actions implemented across Europe. The proposed Multi-Actor and Context Aware framework implies that we examine actions that target (i) different players of the community, that is, the children and adolescents themselves, their families and peers, educational personnel, food suppliers, etc. (ii) different places of interventions (in school, out-school activities, in individuals' homes, at athletic grounds, etc.), (iii) using a set of different means (food regulation, physical activity and exercise, psychological support, utilization of ICT tools etc.).

### 1.1 State-of-the-art description

#### 1.1.1 Pediatric obesity and cancer risk in adulthood

Current epidemiological studies show a strong association between overweight/obesity in childhood and adolescence and an increased risk of several malignancies in adulthood (10). Several mechanisms link childhood obesity to elevated cancer risk, including insulin-like growth factor signaling, chronic low-grade inflammation and oxidative stress, and alterations in adipocytokine pathophysiology (15). However, there remains limited quantification of how individual weight indices (e.g., BMI, waist circumference [WC], waist-to-hip circumference [WHC]) contribute to the carcinogenesis of specific malignancies, and few epidemiological studies establish the extent to which weight control during childhood and adolescence reduces overall adult cancer risk (16). Likewise, comorbidities and related clinical disorders remain underexplored in the context of obesity, with only a few exceptions. (17–19).

#### 1.1.2 Primary interventions for weight control during childhood and adolescence and respective barriers

The current primary interventions for weight management in childhood/adolescence have been mainly focused on controlling one parameter affecting obesity (e.g., healthy food) and therefore they lack from a multi-dimensional assessment across different attributes (20–22). Additionally, the results have been verified only within a small-controlled people space (e.g., within a school) failing to provide concrete conclusions across different countries of diverse socio-economical, cultural and political characteristics and thus of different primary healthcare structures.

### 1.2 Our contribution

In this paper, we assess national programmes for fighting obesity in children and adolescents for fourteen European countries. The assessment target a multi-actor and multi-space framework using different types of means. The analysis concludes main statistical results per national programme and reveal its limitations and barriers. Structural and implementation limitations are examined, modifications in educational curricula and constraints of the educational system itself. Family engagement measures are also investigated along with implementation barriers due to community restrictions, cultural beliefs, and family income status. The analysis is finally completed with financial barriers and economic constraints.

While childhood obesity contributes to a broad range of adult diseases—including type 2 diabetes, cardiovascular disease, and fatty liver disease—this review focuses specifically on cancer. This focus reflects the aim of the PREVENT project, within which this work is conducted: the primary prevention of cancer through the control of childhood obesity (23). The interventions assessed here act on obesity directly, so their relevance extends beyond cancer to the broader burden of obesity-related disease; we therefore frame the comparison around cancer while recognizing these wider benefits. Accordingly, in Section 2 we first establish childhood obesity as a modifiable cancer risk factor in adulthood. Section 4 then describes each national programme, summarizing its coverage, principal results, current status, and limitations. Section 5 provides an overall assessment, covering the financial and economic constraints that hinder wide implementation, educational-system restrictions, and cultural, family, and peer barriers.

## 2 Childhood obesity as a causal, modifiable cancer risk factor in the adulthood

Current clinical studies show that obesity (or even overweight) in childhood and adolescence is associated with increased morbidity and mortality in adulthood, including a higher risk of cancer (24). In Europe, the latest WHO Childhood Obesity Surveillance Initiative (COSI) reports indicate that approximately one quarter of children aged 7–9 years live with overweight and about one in ten with obesity. Post-pandemic trends show stagnation or worsening in many countries (25). Early-life adiposity is linked not only to adult obesity but also to subsequent cancer risk and mortality, strengthening calls for integrated, systemic interventions that go beyond individual behavior to reshape obesogenic environments (26–28).

**Figure 1:**
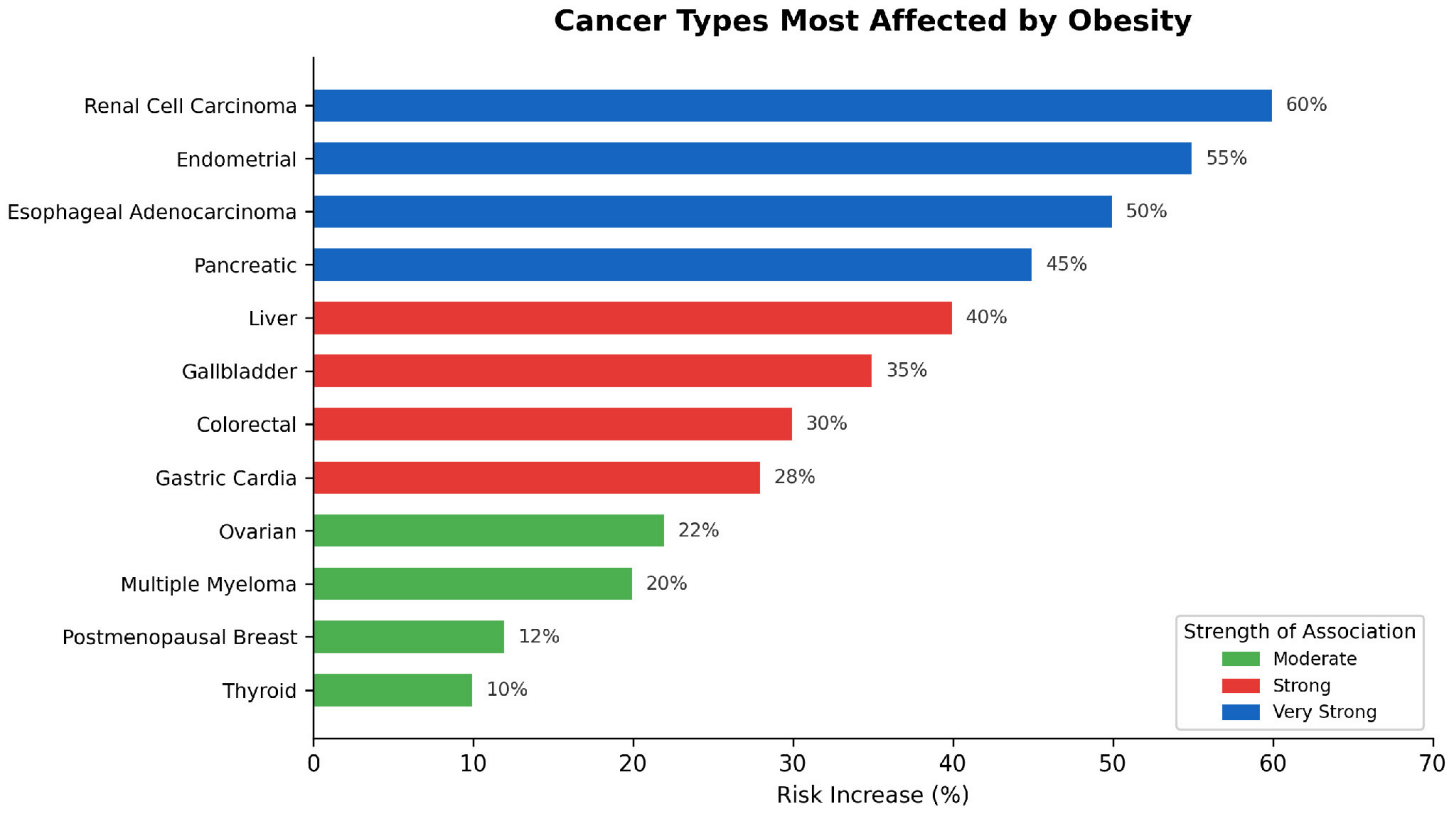
The contribution of obesity in different types of cancer [sources: IARC Working Group (30), Recalde et al. (31, 32)].

This evidence draws on accumulating data from large cohorts, pooled analyses, and meta-analyses. The IARC evaluation extended causal attribution to at least 13 cancer types, including colorectal, endometrial, postmenopausal breast, renal cell carcinoma, pancreatic, liver, gallbladder, gastric cardia, esophageal adenocarcinoma, ovarian, thyroid, meningioma, and multiple myeloma, among others (29, 30). These associations are shown in Figure 1. Subsequent large-scale studies have broadened the map of adiposity-associated malignancies by leveraging longitudinal exposures, refined adiposity metrics, and metabolically defined phenotypes. For example, a multi-cohort, population-based analysis in Catalonia, Spain, examined longitudinal BMI constructs, including age of onset, duration, and cumulative exposure to overweight and obesity, in a population of over 2.6 million adults, demonstrating that longer exposure and earlier onset significantly increase risk across a broader set of cancers than captured by a single baseline BMI measure (31). This work showed positive associations with corpus uteri, kidney, gallbladder and biliary tract, postmenopausal breast, leukemia, multiple myeloma, colorectal, liver, thyroid, and central nervous system cancers, while also adding signals for non-Hodgkin lymphoma, malignant melanoma, prostate, and bladder, particularly when restricting to never-smokers for head and neck and bladder cancers (32, 33).

Cancer risk is also linked to BMI in adolescents. More specifically, cancer risk rises significantly when BMI exceeds the 75th percentile during adolescence, and further above the 95th percentile; the hazard ratio is roughly 2.5 times higher for adolescents above the 95th percentile, indicating a substantially elevated cancer risk that persists into adulthood (10). This underscores the importance of early intervention during childhood and adolescence. Metabolic obesity phenotypes add further granularity: a systematic review and meta-analysis of cohort studies evaluating metabolically healthy and unhealthy normal-weight, overweight, and obese phenotypes reported that metabolic dysfunction independently increases risk across a spectrum of malignancies, and that the combination of adiposity with metabolic dysfunction carries the highest risks (34).

Beyond incident risk, adiposity affects subsequent outcomes and survivorship. Higher BMI at diagnosis relates to elevated risks of second primary cancers and can influence recurrence, treatment tolerance, and toxicity profiles. For instance, dose–response evidence suggests an increased risk of chemotherapy-induced peripheral neuropathy with higher BMI, especially in regimens using taxanes or platinum agents, highlighting how adiposity intersects with oncology care delivery and adverse event profiles (35, 36).

## 3 The European childhood-obesity landscape

### 3.1 Scope, selection criteria, and comparative measures

This work is a comparative policy review rather than a systematic review. Programmes were selected purposively to reflect the diversity of national and regional approaches to preventing childhood and adolescent obesity across Europe, including both school-based interventions and complementary structural and fiscal policies that shape the food environment around schools. The aim was breadth of policy design and implementation context across the continent, rather than exhaustive coverage of every initiative in every country.

Countries and programmes were included in this review if they met the following criteria: (i) a European country with at least one nationally or regionally significant obesity-prevention strategy targeting children and adolescents; (ii) a strategy with either a school-based component or a complementary structural or fiscal policy; and (iii) sufficient publicly available documentation—programme or ministry reporting, surveillance data, or peer-reviewed evaluation—to characterize its design and status and, where available, its reported outcomes. Initiatives were excluded if no implementation details or outcome information could be obtained, or if they targeted adults exclusively. To capture the continent's structural and socio-economic heterogeneity, the selection deliberately spanned the Nordic, Western, Central, Southern/Mediterranean, and Eastern European regions, as well as a range of intervention types, from universal school-meal provision and multi-component school programmes to national surveillance systems, community-based models, fiscal policies, and research-driven living-lab approaches. On this basis, fourteen countries with adequate documentation were retained: Austria, Belgium, Denmark, Finland, France, Germany, Greece, Hungary, Italy, the Netherlands, Portugal, Spain (Catalonia), Sweden, and the United Kingdom. Programme identification and selection were carried out collaboratively by the authors. Rather than analyzing primary data, this review compiles and compares findings reported in peer-reviewed publications and programme evaluations, which are cited in the country-level descriptions in Section 4. The review didn't set a specific start date but looked at strategies used over roughly the past twenty years, from around 2005 to 2025. It included long-standing universal programs that are still active today, like Finland's free school meal program, which has been running since 1948. The focus was mainly on the latest available evaluation evidence for each strategy.

Throughout the comparison, we use a small set of summary measures, defined here before they are applied in Sections 4, 5. *Programme reach* is the number of children or students who directly participate in a school-based programme; for population-level fiscal policies, where no student denominator applies, reach is instead reported as an estimate of the school-age population potentially affected, recognizing that the dietary impact is indirect and mediated by parental purchasing. Therefore, they are reported separately rather than combined. *Programme coverage* (%) denotes the share of the eligible child population reached by a given strategy. *Per-child investment* (€/year) expresses reported programme budgets relative to the population covered; compiled from national programme reports and Eurostat education statistics (37) of differing granularity, it is intended as an order-of-magnitude indicator rather than a precise accounting figure. The *policy-framework score* (0–10) is an author-derived composite summarizing the strength of a country's policy environment. It was assigned by rating each country against the policy domains of the NOURISHING (38) and MOVING (39) frameworks—food standards in public institutions, economic and affordability tools, marketing restrictions, school physical-activity provision, active-transport infrastructure, and public communication—informed by the CO-CREATE policy assessment (40). It is intended as a comparative ordering of the countries reviewed here rather than a validated instrument, and does not reproduce the published NOURISHING or MOVING policy index values. Childhood-obesity prevalence figures are based on WHO COSI surveillance (25); because reported prevalence varies with the age band and BMI cut-off applied, the values used here are indicative rather than exact. Where a value represents an estimate or a synthesis of differently reported source figures rather than a single directly reported number, this is indicated at the point of use.

### 3.2 Regional disparities in prevalence, coverage, and investment

**Figure 2:**
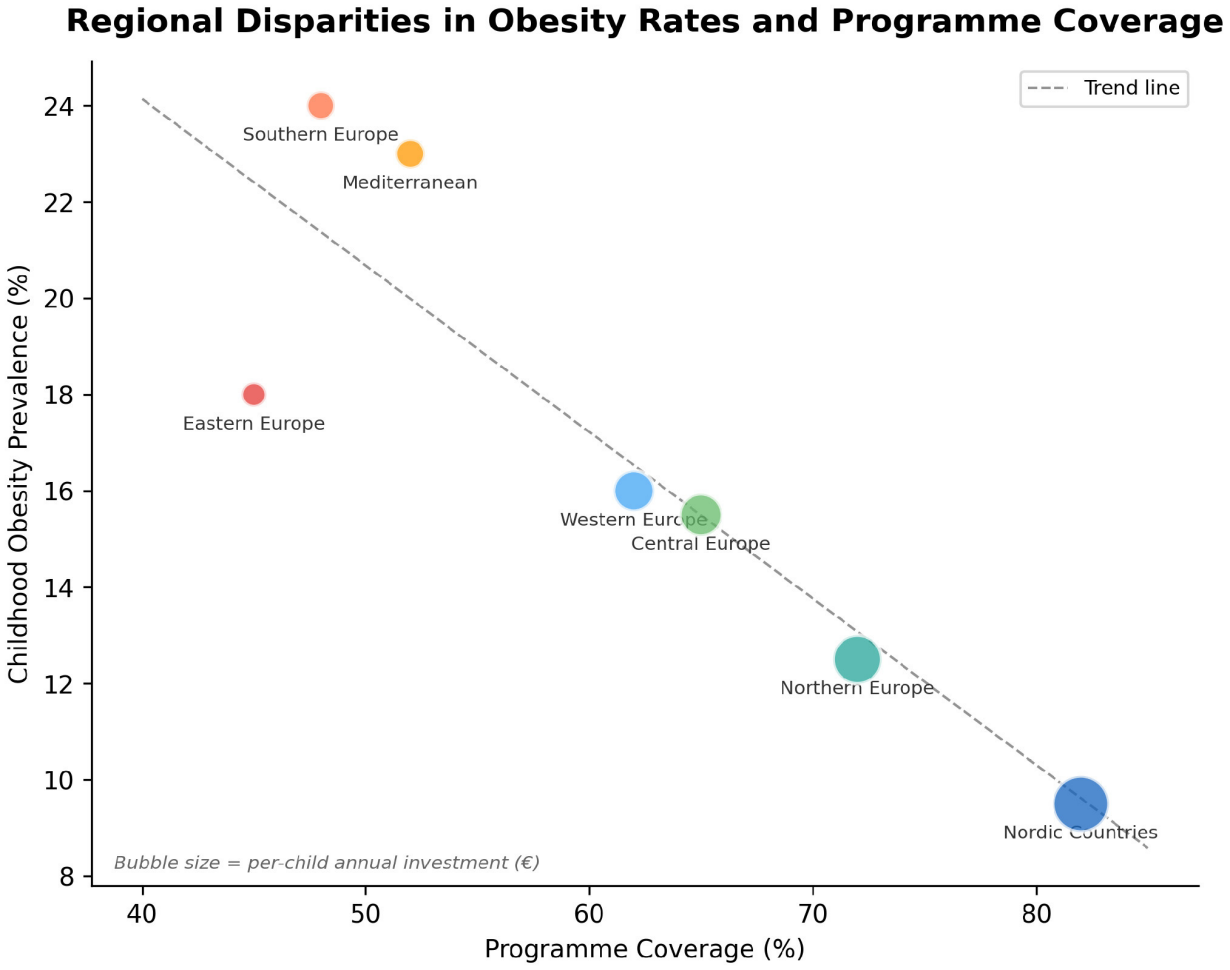
Regional disparities in childhood obesity prevalence, prevention-programme coverage, and per-child investment across European regions. Bubble size = per-child annual investment. Prevalence from World Health Organization Regional Office for Europe (25); enrolment denominators from Eurostat (37). Coverage and per-child investment are the authors' synthesis of national programme reports and are intended as order-of-magnitude indicators, not directly reported figures.

Childhood-obesity prevalence varies markedly across European regions, as do the resources countries direct toward prevention. Prevalence figures are drawn from WHO COSI surveillance (25) and coverage from Eurostat education statistics (37), whereas per-child investment is compiled from national programme reports and, as set out in Section 3, is intended only as an order-of-magnitude indicator; the regional aggregates reported here are the regional aggregates reported here are the authors' order-of-magnitude estimates rather than directly measured statistics. Prevalence is lowest in the Nordic countries (Norway, Sweden, Finland, and Denmark), at around 9%, where programme coverage is highest (approximately 82%) and per-child investment greatest (on the order of €340/year), supported by universal school-meal provision (in Finland since 1948) and health promotion integrated with school services. Mediterranean regions show the highest burden (around 24%) alongside the lowest coverage (approximately 48%) and investment (roughly €88 per child per year), with pronounced within-country gradients—parts of southern Italy exceed 40% while northern regions remain near 20%. Eastern Europe occupies a comparable position (around 18% prevalence, 45% coverage, €65 per child), and Western and Central Europe fall in between, with substantial internal variation. These regional patterns are summarized in Figure 2. Whether higher coverage and investment translate into lower prevalence is examined in Section 5.

### 3.3 Policy frameworks and their strength

The European Union Action Plan on Childhood Obesity 2014–2020 laid the foundation for coordinated national efforts, emphasizing that schools can reach nearly 100% of children across diverse ethnic and socioeconomic backgrounds through structured interventions targeting nutrition education, physical activity enhancement, and environmental modification (41–43).

Policy strength nonetheless varies markedly across nations, with countries such as the Netherlands, Norway, and Portugal exhibiting more robust frameworks than others. The CO-CREATE assessment of five countries (the Netherlands, Norway, Portugal, Poland, and the UK) found that the most successful policies combine the six domains defined in Section 3: healthy food standards in public institutions, economic tools addressing affordability, restrictions on food advertising to children, school-based physical activity, active-transport infrastructure, and public communication (40). Countries with stronger frameworks tend to act across all six NOURISHING (38) and MOVING (39) domains, whereas weaker frameworks show gaps in areas such as advertising restrictions or active transport. COSI surveillance indicates that countries with more comprehensive school-based policies tend to record lower childhood obesity prevalence, although implementation challenges persist (25).

**Figure 3:**
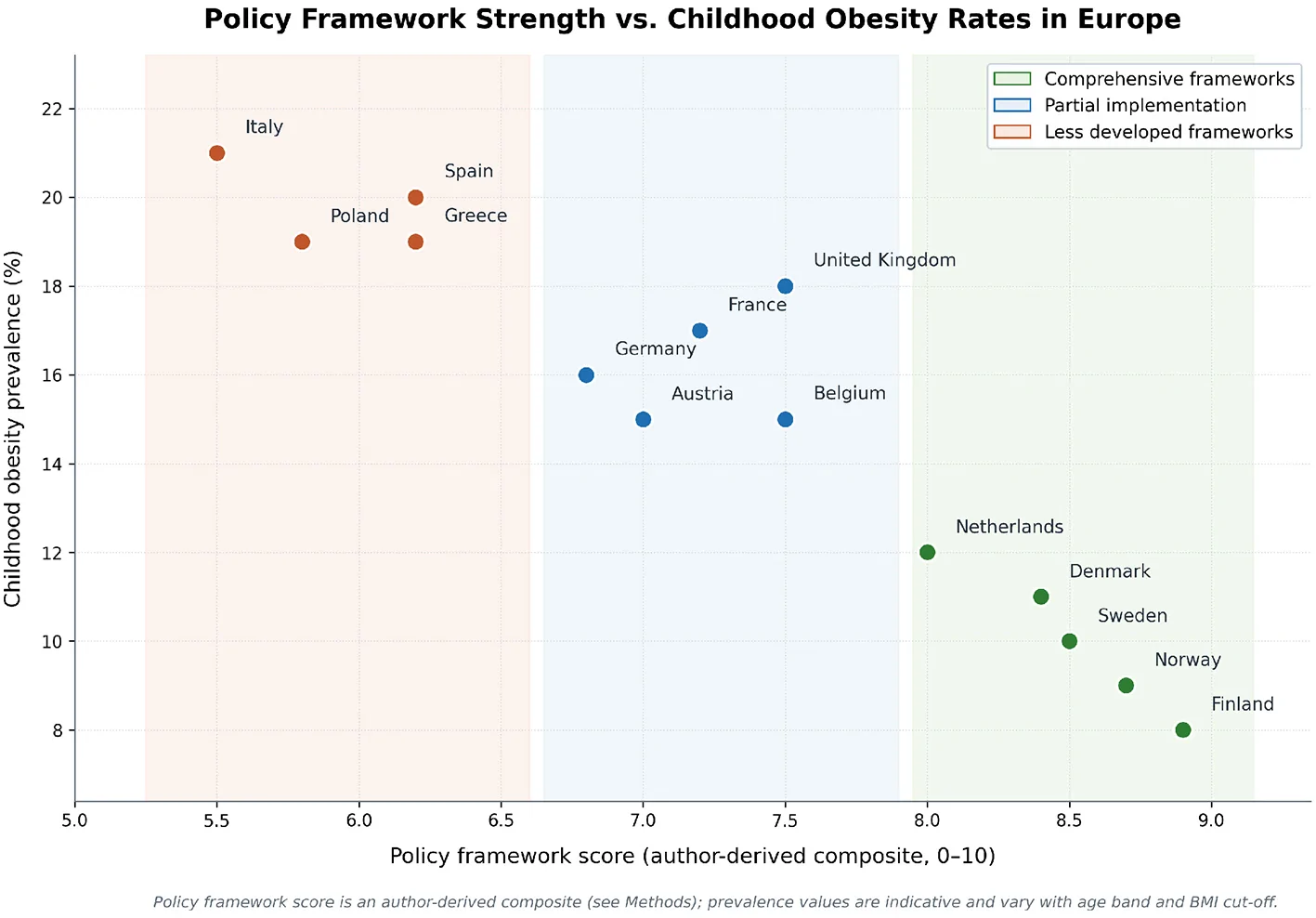
Policy framework strength and childhood obesity prevalence across European countries. The policy framework score is an author-derived composite (see Section 3) intended as a comparative ordering rather than a validated index; prevalence values are indicative and vary with the age band and BMI cut-off applied (25). The association shown is ecological and descriptive and should not be interpreted causally.

This pattern is summarized in Figure 3, which positions countries by policy-framework strength against childhood-obesity prevalence. Countries with the most comprehensive frameworks—Finland, Norway, Sweden, Denmark, and the Netherlands—act across all six domains and record the lowest prevalence among those reviewed. Middle-tier countries, including Germany, Austria, France and the United Kingdom, show partial implementation with notable gaps: France's PNNS (44) has been associated with stabilization of prevalence in some regions alongside persistent socioeconomic disparities, while the UK's Childhood Obesity Plan reduced sugar consumption through the Soft Drinks Industry Levy without a corresponding fall in prevalence. Countries with less developed frameworks, including Greece, Poland, Spain and Italy, face coordination challenges, limited binding enforcement, regional resource disparities, and inadequate support for high-risk populations (25). This relationship is ecological and descriptive: reported prevalence varies with the age band and BMI cut-off applied, and the association cannot be interpreted causally, since policy strength co-varies with national income, health-system capacity and other structural factors.

## 4 National school-based obesity prevention strategies in Europe

In the following, we briefly describe the major obesity programmes in 14 European countries along with the man limitations and advntages of each programme. Table 1 shows a summary of the programmes per country.

**Table 1:** Summary of national and regional school-based obesity prevention strategies in Europe.

Country	Initiative	Type	Key components	Status	Limitations	Results
Austria	National Nutrition Action Plan (NAP.e)/EDDY Project	National strategy + school-based intervention	Multi-sectoral coordination; school nutrition guidelines; EDDY project (10 h per semester nutrition + PA education); teacher training; parental engagement	NAP.e ongoing since 2011; EDDY pilot 2022–2024 in Vienna schools; national obesity treatment system development	Implementation coordination challenges; limited binding enforcement; regional resource disparities; summer-period intervention gaps	EDDY results: stable BMI-SDS, improved quality of life and motor skills; mixed population-level outcomes
Belgium	Oog voor Lekkers (EU School Scheme, Flanders)/School Fruit Program	School-based food provision	Subsidized fruits, vegetables, and milk distribution; educational activities; nutritional goals; obesity prevention focus	Ongoing since EU School Scheme implementation, targeting schools with >15% disadvantaged students	Limited to snack provision only; insufficient curriculum integration; regional disparities in implementation quality; minimal family engagement beyond school setting	Increased enrolment in participating schools; impact evaluation ongoing (results pending 2023–2024)
Denmark	School Meal Pilot Scheme/National School Food Standards	School-based universal program	Free and subsidized school meals; nutritional guidelines; educational components; portion-size standards; food quality regulations	National pilot 2025–2029 (DKK 854 million funding); 191 schools participating in evaluation phase	Recent implementation limiting evaluation; cost sustainability concerns; limited integration with broader health promotion; municipal implementation variations	Too early for comprehensive evaluation; promising framework based on Nordic model precedents
Finland	Free School Meal Programme	Universal school-based food provision	Universal free meals; nutritional standards; food education integration; municipal implementation; regular quality monitoring	Established 1948; universal coverage achieved; continuous updates and quality improvements	Municipal implementation variations; budget constraints affecting quality; limited family habit connection; diverse dietary accommodation challenges	Modest obesity prevention impact; high participation rates; strong nutritional quality maintenance
France	National Nutrition and Health Program (PNNS)	National comprehensive strategy	Multi-sectoral coordination; media campaigns; healthcare integration; school programs; regional adaptation; research support	Fifth cycle (PNNS 5), launched in 2026; continuous evolution since 2001; regional project variations	Persistent socio-economic disparities; limited high-risk population effectiveness; insufficient component integration; difficult long-term impact measurement	Stabilization of childhood obesity prevalence in some regions; variable effectiveness across population groups
Germany	IN FORM – National Action Plan/Guidelines to Prevent Obesity	National strategy framework	Physical activity mandates (60–90 min/day); quality standards for school meals; media consumption limits; teacher training; parental engagement	Guidelines established 2016, non-binding implementation across Länder with significant regional variation	Non-binding nature of guidelines; regional implementation disparities; insufficient funding for nationwide rollout; limited long-term follow-up data	KOPS study showed some favorable effects in intervention schools; limited population-level impact documentation
Greece	PREVENT Project/National Health Policies	Research project + national framework	Living labs methodology; co-creation approach; digital tools; multi-actor interventions; AI-powered analytics	PREVENT active 2022–2025; national policy framework development	Limited geographic scope; high complexity; resource requirements; technology dependency; sustainability planning challenges	Research project results pending; innovative methodology development; limited population coverage
Hungary	Public Health Product Tax (PHPT)	Comprehensive fiscal policy	Multi-category unhealthy food taxation; regular legislative updates; revenue hypothecation for health; wide product coverage	Implemented 2011; multiple amendments; ongoing evaluation and adaptation	Frequent legislative amendments needed; industry circumvention; administrative complexity; limited population health impact evidence	Short-term reduction in consumption of taxed products, not sustained over time; revenue generation for health sector; limited obesity prevalence impact
Italy	OKkio alla SALUTE/Integrated Health Campaigns	Surveillance + communication strategy	National surveillance system; public health campaigns; school-based activities; Olympic sport promotion; regional coordination	Ongoing since 2008; regular reporting cycles; integration with WHO-COSI	Limited surveillance-to-intervention translation; regional implementation disparities; minimal healthcare system integration; campaign sustainability issues	Comprehensive surveillance data; variable intervention implementation; limited population-level impact documentation
Netherlands	JOGG (Youth at Healthy Weight) – EPODE adaptation	Community-based integrated approach	Multi-stakeholder collaboration; local policy changes; community activities; environmental interventions; municipal coordination	Active in 170+ municipalities since 2010 (>2/3 of Dutch municipalities participating)	Mixed effectiveness results; implementation quality variations; limited impact on BMI and physical activity; challenging evaluation methodology	Modest improvements in fruit/vegetable consumption; no significant BMI reduction; some evidence of effectiveness in low-SES areas long-term
Portugal	Sugar Tax/School Health Promotion	Fiscal policy + school programs	Tiered sugar-sweetened beverage tax; school health promotion; product reformulation incentives; consumption monitoring	Tax implemented 2017; ongoing assessment and adaptation	Limited scope to beverages; potential regressive effects; cross-border shopping; insufficient complementary interventions	21% reduction in SSB sales; significant product reformulation; estimated 40–78 obesity cases averted annually
Spain	Regional programs (Catalonia)/PREVENT Project participation	Regional strategy + research project	School food restrictions; regional health promotion; PREVENT living labs methodology; digital engagement platforms	Regional variations; PREVENT active 2022–2025; pilot implementations	Regional implementation disparities; limited national coordination; complex multi-dimensional approach challenges; resource intensity	Variable by region; PREVENT results pending; some evidence of regional program effectiveness
Sweden	Healthy School Start/National Physical Activity Guidelines	Universal school-based health promotion	Parental motivational interviewing; health information materials; classroom activities; teacher training; multi-behavioral focus (diet, PA, screen time, and sleep)	Multiple RCT evaluations 2011–2020; integrated with school health services; universal coverage in participating areas	Limited sustained effects; requires intensive implementation; gender-specific differential effects; need for program intensification	Improved vegetable intake in children; some gender-specific PA improvements; no significant obesity prevalence reduction
United Kingdom	Childhood Obesity Plan	National multi-component strategy	Soft drinks industry levy; reformulation targets; marketing restrictions; school food standards; physical activity promotion; calorie labeling	Launched 2016, updated 2018/2019; ongoing implementation with variable compliance	Industry resistance; limited enforcement; insufficient socio-economic focus; policy continuity challenges; inadequate high-risk population support	Some reduction in sugar consumption; limited obesity prevalence impact; industry reformulation achieved

**Figure 4:**
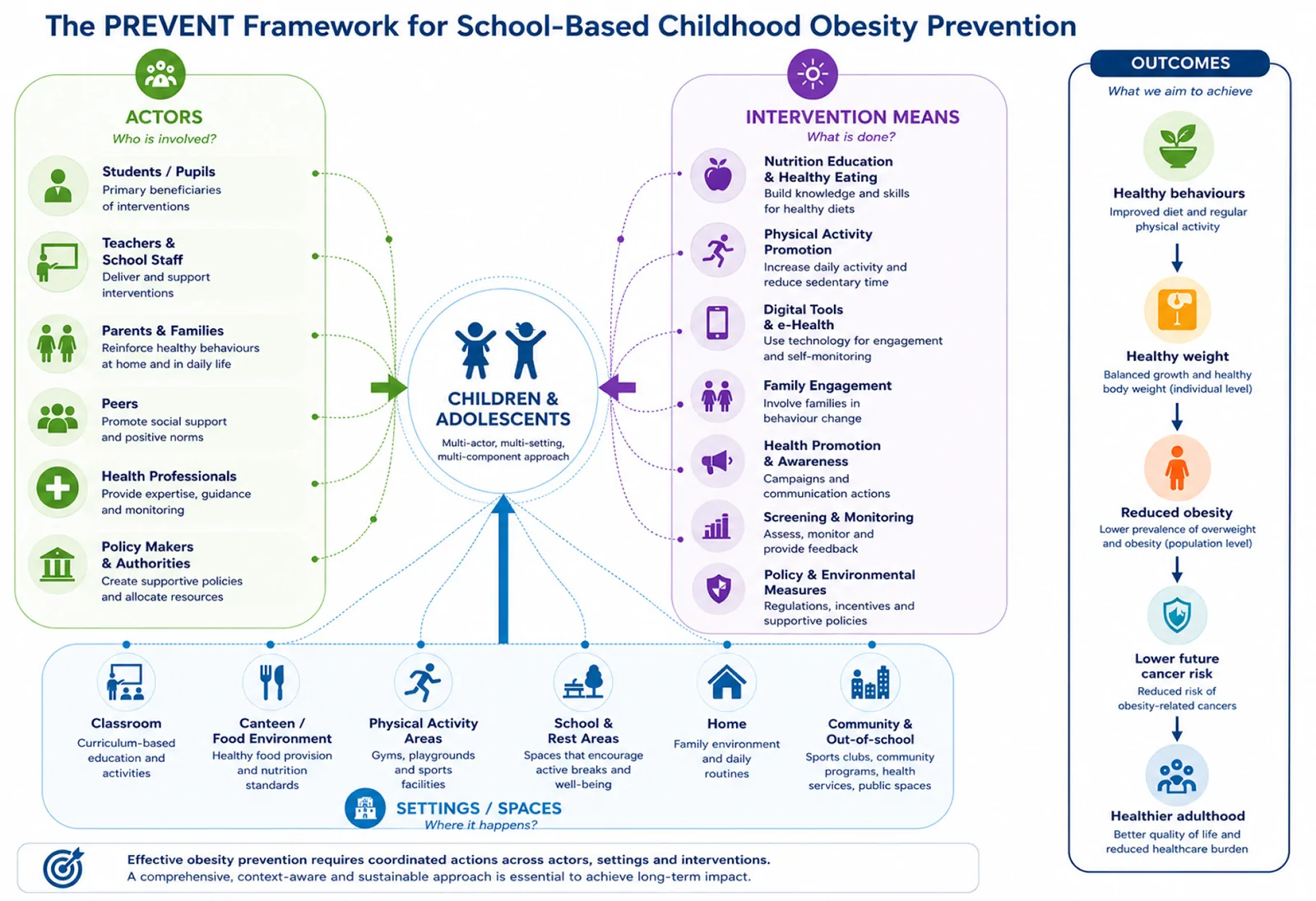
Conceptual framework of the PREVENT approach to school-based childhood obesity prevention, organized around the socio-ecological model. The framework links actors (who is involved), intervention means (what is done), and settings/spaces (where it happens), all converging on children and adolescents, to a chain of intended outcomes—healthier behaviors, healthy weight, reduced obesity, and lower future cancer risk. The framework is author-constructed to organize the intervention components reviewed in this paper; it is not based on empirical measurement.

Across these countries, school-based interventions operate across a range of settings, actors, and tools. Figure 4 maps this diversity: interventions operate in different spaces (classrooms, canteens, and rest areas, among others), engage different actors (pupils, teachers, parents, and peers), and deploy varied means, including digital tools, family engagement, awareness campaigns, and nutrition-profiling activities.

### 4.1 Austria's case

Austria's multi-pronged approach combines the National Nutrition Action Plan (NAP.e), in place since 2011 (45), with the more recent school-based EDDY programme (*Effect of sports and diet training to prevent obesity and secondary diseases and to influence young children's lifestyle*), conducted in primary schools in Vienna (46). The NAP.e framework emphasizes multi-sectoral coordination and promotes action across the education, public health, and community sectors.

The EDDY intervention stabilized participating children's BMI standard-deviation scores (BMI-SDS), preventing the age-related increases observed in comparison groups, without producing absolute BMI reductions, a pattern consistent with normal growth. Analyses used mixed-effects models that accounted for repeated measures and clustering within schools (*p* < 0.05) (46).

Reflecting wider European recognition of obesity as a chronic disease requiring sustained action across the life course (47), Austria is also developing a national obesity-treatment system to complement prevention efforts, acknowledging that prevention alone may be insufficient without structured support for children already living with obesity.

### 4.2 Belgium's case

Belgium's school-based initiative focuses primarily on the subsidized distribution of fruit, vegetables, and milk within educational settings, targeting schools with more than 15% disadvantaged students to address socioeconomic disparities in access to healthy food. It operates within the framework of the European Union School Scheme and incorporates educational activities alongside nutritional goals, with obesity prevention as a stated objective. However, the initiative remains largely confined to snack provision without full integration into the broader curriculum, limiting opportunities for sustained behavioral change beyond the immediate consumption context. These efforts are set against a substantial national burden of overweight, which is a major contributor to non-communicable disease in Belgium (48).

Rigorous impact-evaluation data specific to the scheme's anthropometric and long-term behavioral outcomes remain limited, so its direct effect on childhood obesity cannot yet be firmly established.

### 4.3 Denmark's case

Denmark's principal recent effort is Generation Healthy Kids (GHK), a multi-setting, multi-component obesity-prevention programme for children aged 6–11, developed using a whole-systems approach. National stakeholders were engaged through structured group model-building workshops to map the drivers of childhood obesity and identify priority actions across six subsystems: family; diet and dietary habits; physical activity and active living; mental health and wellbeing; screen, media, and sleep; and the competencies of professionals (49). The resulting intervention spans schools, after-school clubs, families, and local communities, with fixed elements including a school-lunch programme and regular school-based physical activity alongside co-created local components.

GHK is being evaluated as a cluster-randomized controlled trial across schools in three Danish regions, conducted during 2023–2025, with change in children's fat mass as the primary outcome. Effectiveness results are pending completion and analysis of the trial (50).

### 4.4 Finland's case

Finland's universal Free School Meal Programme represents the longest-standing national school meal initiative in Europe, established in 1948 and achieving universal coverage with continuous quality improvements and updates to nutritional standards. The program provides free meals to all students regardless of socioeconomic status, incorporates comprehensive nutritional standards based on Nordic nutrition recommendations, integrates food education into curriculum, operates through municipal implementation with regular quality monitoring, and emphasizes both nutritional adequacy and cultural appropriateness.

However, despite these strengths, Finland has not demonstrated a clear obesity-prevention impact attributable to school meals alone; national childhood obesity trends broadly track those of other Nordic countries, including those without universal meal programmes (25). Reported limitations include municipal variation in meal quality and variety, budget constraints that affect ingredient quality and appeal in some municipalities, a limited connection to family eating habits—school-meal patterns appear not to substantially influence home dietary behavior—and challenges in accommodating diverse dietary requirements related to allergies, religious practices, and ethical preferences.

### 4.5 France's case

France's comprehensive National Nutrition and Health Programme (PNNS) (44) represents one of Europe's longest-standing obesity-prevention strategies, evolving continuously through successive cycles since its initial implementation in 2001. The fourth cycle (2019–2023) was subsequently extended and followed by a fifth cycle (PNNS 5), launched in 2026 (51). Across its iterations, the multi-sectoral programme has coordinated media campaigns, healthcare-system integration, school-based interventions, regional adaptation mechanisms, and research support, creating a comprehensive societal approach to obesity prevention.

A persistent challenge is the strong socioeconomic and territorial gradient in nutrition-related health: obesity prevalence remains higher among children from lower-income and disadvantaged groups, an inequality that PNNS 5 explicitly identifies as a continuing priority for action (51).

### 4.6 Germany's case

Germany's comprehensive national strategy framework, established in 2016, represents an ambitious attempt to standardize obesity prevention approaches across the federal system through non-binding guidelines that mandate 60–90 min of daily physical activity, establish quality standards for school meals, implement media consumption limits, and promote teacher training alongside parental engagement. Germany also has a long tradition of school-based prevention research, exemplified by the Prevention Education Program (PEP), initiated in Nuremberg in 1994 (52).

The Kiel Obesity Prevention Study (KOPS) (53), conducted within intervention schools, demonstrated some favorable effects on anthropometric measurements and health behaviors among participating children. However, the absence of binding enforcement mechanisms, coupled with insufficient funding for comprehensive nationwide rollout, has constrained the initiative's capacity to achieve measurable population-level impact. The lack of long-term follow-up data further complicates the assessment of sustained behavioral changes and obesity prevalence reduction beyond the immediate intervention period. Statistical analyses from KOPS revealed modest improvements in dietary quality and physical activity levels, yet these improvements failed to translate into significant body mass index (BMI) reductions at the population level, suggesting that structural and environmental factors outside the school setting may attenuate intervention effects (54).

### 4.7 Greece's case

The national effort to address obesity in Greece is being implemented through two main initiatives. The first, launched in November 2023, is a comprehensive nationwide programme that spans primary, secondary and tertiary prevention. It targets all children aged 0 to 17 and their families across home, school, healthcare, and community settings.

The overarching aim of the programme is to reduce risk factors and socio-economic disparities linked to childhood and adolescent obesity, preventing long-term chronic conditions through increased physical activity and healthy eating. The primary goal is to reduce obesity in children aged 2 to 14 from 37.5% in 2019 to 24.5% by 2025. The second strategy in Greece is as a part of PREVENT EU funded project (23). The Greek PREVENT implementation emphasizes living labs methodology creating structured environments for co-creation between researchers, practitioners, families, and communities, comprehensive digital tools including mobile applications and AI-powered analytics enabling personalized intervention adaptation based on individual response patterns and risk profiles.

However, the initiative faces significant implementation challenges including limited geographic scope restricted to pilot communities rather than achieving national coverage. Quantified results remain pending given the project's active implementation phase extending through 2025, with comprehensive evaluation anticipated following intervention completion. Greece has also hosted school-based research interventions such as the CHILDREN study, which applied the Theory of Planned Behavior to improve access to physical activity and increase fruit and vegetable availability while strengthening parental support (55).

### 4.8 Hungary's case

Hungary's Public Health Product Tax (PHPT), implemented in 2011, represents one of Europe's most comprehensive fiscal policies targeting unhealthy foods, extending beyond beverages to multiple categories of products high in sugar, salt, and caffeine (56). It was introduced against a backdrop of high obesity prevalence: a large primary-care evaluation found overweight in around 40% of men and 31% of women, and obesity in roughly 32% of both (57).

Longer-term evidence indicates limited durable impact. A longitudinal analysis of household purchase data found that, although the tax initially reduced consumption of taxed products, this effect was offset over time: as dietary habits and the food environment did not change and disposable income rose, consumption recovered—and for some categories increased—while the tax was largely passed through to retail prices (56). The authors conclude that taxation alone is insufficient to produce durable reductions in consumption and may widen social inequalities, since price rises fall hardest on lower-income households; sustained change instead requires complex, multi-component interventions addressing dietary habits and the food environment alongside fiscal measures. Consistent with this, childhood-obesity prevalence in Hungary has not shown a clear decline under the tax (25).

### 4.9 Italy's case

Italy's OKkio alla SALUTE initiative, operational since 2008, combines a comprehensive national surveillance system with integrated public health campaigns delivered under the Guadagnare Salute national programme, school-based activities, and regional coordination mechanisms (58, 59).

However, the initiative highlights the gap between surveillance and intervention: comprehensive monitoring has not consistently translated into evidence-based action at regional and local levels. Childhood overweight and obesity remain markedly higher in southern regions, reflecting a persistent north–south gradient and underlying socioeconomic disparities, despite sustained surveillance and awareness activities (59).

### 4.10 The Netherlands' case

The Dutch JOGG initiative (*Jongeren Op Gezond Gewicht*), the national adaptation of the EPODE model, has been implemented across more than 170 municipalities since 2010, employing a community-based integrated approach (60).

An area-level analysis mapping anthropometric data for over 200,000 Dutch children found that overweight prevalence in JOGG areas fell from 25.2% in 2013 to 16.1% in 2018, with the decline concentrated in low socioeconomic status (SES) areas that had implemented the approach for the longer evaluation period (approximately 6 years) (60). However, JOGG areas—which began with substantially higher overweight—remained consistently more affected than non-JOGG areas throughout the period (around 12.6% in 2018), and the study's ecological, repeated cross-sectional design precludes firm causal attribution. These findings suggest that sustained, multi-year implementation in high-need communities is associated with reductions in overweight, while underscoring that JOGG alone did not close the gap with lower-prevalence areas and that the broader evidence on EPODE-style programmes remains mixed.

### 4.11 Portugal's case

Portugal implemented a tiered sugar-sweetened beverage (SSB) tax in 2017, one of Europe's earlier fiscal interventions targeting obesity prevention, complemented by school health-promotion programmes and incentives for product reformulation (61).

Official evaluation data indicate that, in the first year after implementation, SSB sales fell by approximately 7%, while reformulation reduced the total energy intake from sweetened beverages by around 11%; the share of high-sugar drinks (containing at least 80 g of sugar per liter) fell from roughly 61% to 38% of sales volume as manufacturers shifted products into lower-tax tiers (61). Modeling of these combined demand and reformulation effects estimated that the tax could prevent approximately 40 to 78 new cases of obesity per year, with the joint impact of reduced consumption and reformulation several times larger than that of reformulation alone (62).

The Portuguese experience demonstrates that fiscal policies can modify specific consumption behaviors and incentivise industry reformulation; however, the projected population-level effect on obesity incidence remains modest, reinforcing that meaningful reductions likely require comprehensive, multi-component approaches extending beyond single-product interventions (62).

### 4.12 Spain's case

Spain's decentralized governance has led to substantial regional variation in childhood obesity-prevention strategies, with Catalonia developing one of the country's most comprehensive regional approaches. A prominent example is the school-based EdAl (*Educació en Alimentació*) programme in the Reus area, a nutrition- and lifestyle-education intervention delivered in primary schools by trained university students acting as “health-promoting agents” over three academic years (63, 64). Catalonia also participates in newer research initiatives, including the EU PREVENT project (23).

The EdAl trial provides one of the clearest regional impact assessments in Spain. Over the 28-month intervention, obesity prevalence among boys decreased by 2.36% in the intervention group while increasing by 2.03% in controls—a between-group difference of 4.39% (95% CI 3.48–5.30; *p* = 0.01)—together with a favorable reduction in BMI z-score among intervention boys and a higher proportion of pupils undertaking more than five hours of physical activity per week; no significant effect was observed among girls (64). A 2-year post-intervention follow-up suggested that some of these benefits persisted, with lower BMI z-scores and obesity prevalence than in the control group, although the long-term effects were more modest than those observed immediately after the intervention (65). Results for Catalonia's PREVENT component remain pending, with comprehensive outcome evaluation anticipated following the conclusion of intervention delivery in 2025.

The limited national coordination creates missed opportunities for knowledge transfer and standardization of effective approaches across autonomous communities, with successful regional innovations often remaining localized rather than being scaled nationally.

### 4.13 Sweden's case

Sweden's Healthy School Start (HSS) initiative is a school-based health-promotion programme delivered in the preparatory school year (children aged 5–7) through parental support, combining health information, motivational interviewing with parents, and teacher-led classroom activities. It has been evaluated in successive cluster-randomized controlled trials (RCTs) since 2010, providing relatively robust evidence of intervention effectiveness (66, 67).

The first trial, conducted in areas of low-to-medium socioeconomic position, found significantly higher vegetable intake in the intervention group than in controls, together with higher total physical activity among girls at weekends (66). A second trial in disadvantaged, low-SES areas found beneficial effects on the intake of unhealthy foods and drinks and a significant reduction in BMI standard deviation score among children who were obese at baseline, although no significant effect on BMI was observed across the study population as a whole (67). Taken together, these trials indicate that a relatively low-intensity, parental-support intervention delivered through schools can improve dietary behavior and benefit higher-risk children, while its impact on population-level weight outcomes remains modest. A 4-year follow-up of the second trial found that most intervention effects were attenuated over the longer term, although a reduced intake of unhealthy foods persisted among boys, underscoring that sustaining early behavioral gains is challenging without continued support (68).

### 4.14 The United Kingdom's case

The United Kingdom's Childhood Obesity Plan, launched in 2016 with subsequent chapters in 2018 and 2019, is a national multi-component strategy combining fiscal policy, industry regulation, school-based interventions, and environmental measures. Its components include the Soft Drinks Industry Levy (SDIL, commonly called the “sugar tax”), sugar-reduction and reformulation targets for processed foods, restrictions on marketing to children, strengthened school-food standards, physical-activity promotion, and mandatory calorie labeling in the out-of-home sector (69).

Evaluations indicate that the levy operated primarily by prompting manufacturers to reformulate products to lower their sugar content, rather than by reducing the overall volume of soft drinks consumed. A controlled interrupted time series analysis of British household purchasing found that, one year after implementation, the volume of high-sugar (high-tier) drinks purchased fell by more than a third, while the total volume of soft drinks purchased was unchanged, as households shifted toward reformulated, lower-sugar products (69). Reformulation was substantial, with many manufacturers reducing sugar content to bring products below the levy thresholds (70). However, because overall soft-drink volume was unchanged and the levy addresses a single product category, its measurable effect on population-level obesity prevalence remains limited and debated, reinforcing the conclusion that fiscal measures require complementary interventions to influence obesity outcomes.

Beyond country-specific strategies, several multi-country EU initiatives operate across these national contexts. The Erasmus+ EUMOVE project developed school-based resources to promote healthy lifestyles and prevent obesity in children and adolescents across several European countries (71). The ToyBox study, implemented in several European countries, targeted preschoolers' energy-balance-related behaviors—water consumption, healthy snacking, physical activity, and reduced sedentary time; subsequent analyses have examined how compliance with 24-hour movement-behavior guidelines relates to adiposity indicators in this age group (72).

Table 1 shows a summary of the programmes per country.

## 5 Assessment of different European programmes for obesity prevention

Having described the national strategies, we now assess their performance and costs, as well as the structural, educational, and socio-cultural barriers that constrain their implementation.

### 5.1 Performance results of European programmes and investment costs

**Figure 5:**
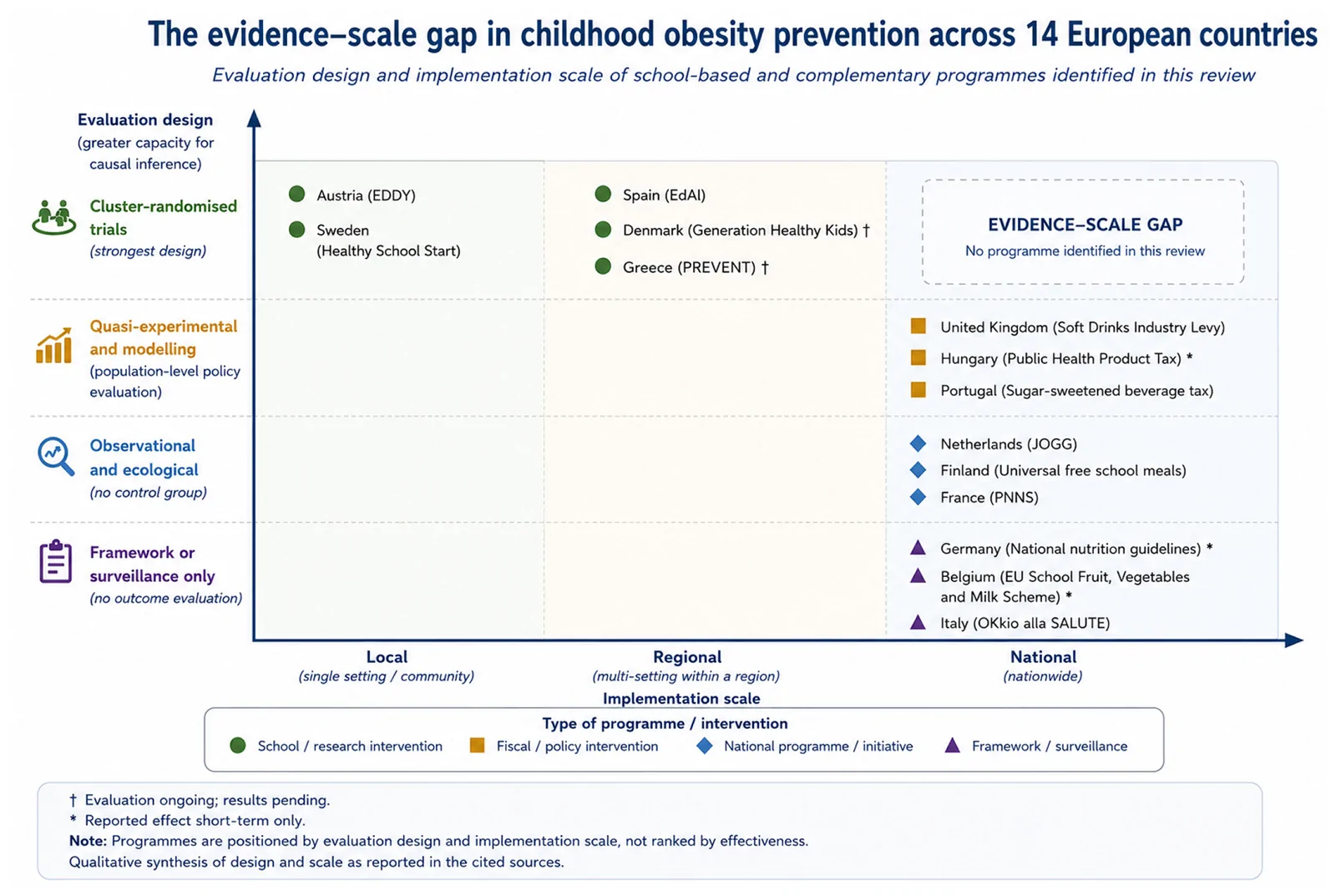
Evaluation design and implementation scale of school-based and complementary childhood obesity-prevention programmes identified across the 14 European countries reviewed. Programmes are positioned by the strongest evaluation design reported in the cited sources (vertical axis, ordered by capacity for causal inference) and by implementation scale (horizontal axis). Programmes evaluated by cluster-randomized trial operate only at local or regional scale, whereas all nationally delivered programmes rest on quasi-experimental, observational, or framework-level evidence—the evidence–scale gap indicated at upper right. The corresponding absence of local and regional programmes in the lower evidence tiers should be interpreted with caution, as small-scale initiatives typically enter the published literature only when formally evaluated. † evaluation ongoing, results pending; * reported effect short-term only. The figure is a qualitative synthesis of design and scale as reported in the cited sources; programmes are not ranked by effectiveness.

Figure 5 summarizes the landscape of school-based obesity prevention across the 14 countries reviewed, positioning each programme by implementation scale and by the strongest evaluation design reported in the cited sources. Three groups emerge. National and universal measures—Finland's free school meal programme, France's PNNS (44), Germany's IN FORM guidelines, the Netherlands' JOGG rollout, and Italy's OKkio alla SALUTE—operate at population scale but rest on observational designs, and OKkio functions as surveillance rather than intervention. Fiscal measures in the United Kingdom, Portugal and Hungary act at population level through pricing, assessed with quasi-experimental or modeling approaches; their dietary effect is indirect and, in the Hungarian case, reported only in the short term. By contrast, the programmes with the strongest designs—cluster-randomized trials including Austria's EDDY, Spain's EdAl, Sweden's Healthy School Start, Denmark's Generation Healthy Kids, and the Greek PREVENT living-lab implementation—operate at pilot or regional scale. This distribution reveals a persistent evidence–scale gap: no nationally delivered programme has yet been evaluated by cluster-randomized trial, while the interventions with the most rigorous evidence have not been scaled nationally. Direct comparison of students reached is not meaningful across these categories, since universal programmes cover entire cohorts by design whereas trial reach reflects a study sample.

Meta-analyses of European school-based interventions demonstrate that multi-component programs combining diet and physical activity interventions produce favorable effects on BMI, biomarkers, eating behaviors, and self-efficacy, with the most successful interventions characterized by theoretical grounding, teacher training, parental involvement, and duration of at least one school year (42, 73). However, systematic reviews reveal that while many interventions show positive short-term effects, long-term sustainability and scalability remain significant challenges, with success rates varying considerably based on implementation fidelity, local adaptation, and ongoing support systems. The emerging European Programme OBELISK represents the next generation of precision-based approaches, aiming to prevent childhood obesity through prediction, prevention, precision medicine, and participatory methods that engage families and communities in comprehensive, personalized interventions based on genetic, environmental, and behavioral risk factors (74).

### 5.2 Structural and implementation limitations

The most pervasive constraint is inadequate funding and resource allocation, with studies consistently identifying limited budgets, insufficient equipment, and lack of appropriate facilities as primary barriers to successful implementation (75, 76). Countries with otherwise robust policy frameworks struggle with implementation gaps, where 60% of European nations have formal obesity prevention strategies yet none are on track to meet WHO targets for halting obesity increases by 2025 (77, 78). The structural barriers extend beyond financial constraints to encompass organizational challenges including time limitations for stakeholders, overloaded school curricula that prioritize academic achievement over health promotion, and insufficient specialist staff in nutrition and physical education (75). Furthermore, the lack of comprehensive monitoring and evaluation systems across European countries prevents adequate assessment of policy effectiveness, creating a cycle where ineffective interventions continue without modification while successful programs cannot be properly scaled or replicated (79).

### 5.3 Curriculum and educational system constraints

The integration of obesity prevention within existing educational frameworks reveals significant limitations stemming from competing priorities and rigid curriculum structures across European school systems. Teachers consistently report that academic performance pressures, standardized testing requirements, and overcrowded curricula leave insufficient time for comprehensive health education and physical activity programming, with physical education often receiving lower priority compared to core academic subjects (75, 80). The redesigned National Curriculum policies in many European countries have inadvertently reduced extracurricular activities that encourage physical activity and health awareness, making it increasingly difficult for schools to implement, sustain, and strengthen obesity prevention interventions (80). Additionally, the lack of adequately trained teachers in nutrition science and physical education expertise creates implementation gaps, where even well-designed policies fail due to insufficient professional development opportunities and limited understanding of evidence-based obesity prevention strategies among educational staff (75, 76). Gender-specific limitations also emerge, with female schools reporting greater barriers including limited space, insufficient resources, and more severe shortages of qualified sports teachers, highlighting how structural inequalities within education systems compound obesity prevention challenges (75).

### 5.4 Family and community engagement barriers

The effectiveness of European school-based obesity prevention policies is severely constrained by persistent barriers to meaningful family and community engagement, creating disconnections between school-based interventions and home environments where children spend the majority of their time. Parents' reluctance to participate in obesity prevention activities, stemming from insufficient knowledge about nutrition and physical activity, time constraints due to work obligations, and financial limitations that prioritize immediate needs over long-term health investments, represents the most frequently reported barrier at the individual level across European studies (76, 80). Socioeconomic disparities exacerbate these challenges, with low-income families facing additional barriers including limited access to healthy foods, reduced opportunities for organized physical activities, and competing priorities that make sustained engagement with school-based programs difficult to maintain (68, 81). Cultural and linguistic barriers further compound engagement challenges in diverse European communities, where intervention materials and communication strategies often fail to account for varying cultural perspectives on body weight, food practices, and physical activity preferences (76). The disconnect between school-based policies and broader community food environments, including easy access to unhealthy foods around schools and aggressive marketing of processed foods targeting children, undermines even the most comprehensive school-based interventions (80).

**Figure 6:**
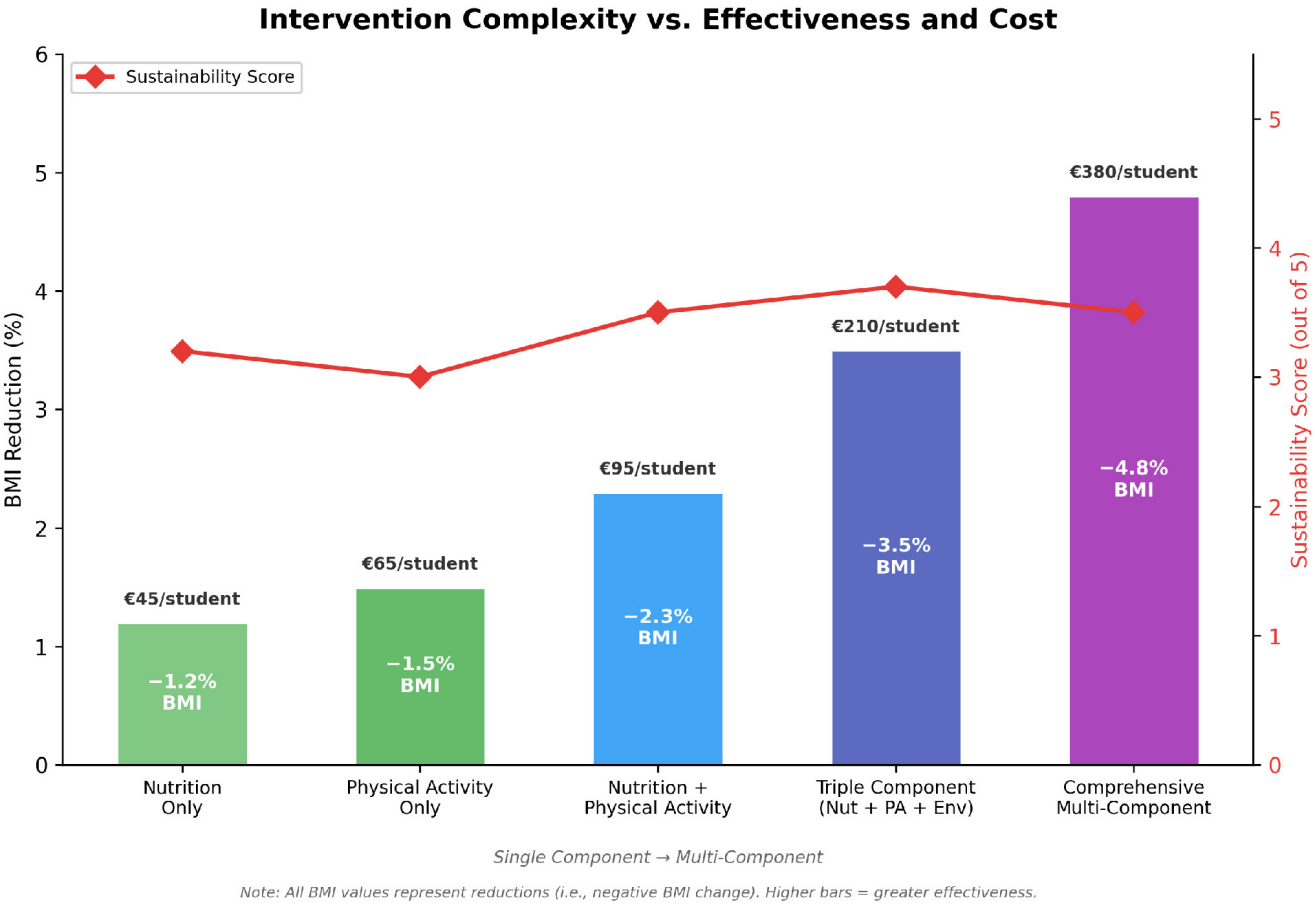
Intervention complexity vs. effectiveness and cost. All BMI values are reductions [sources: Drouka et al. (42) and Spiga et al. (21)].

In Figure 6 we present a plot that shows objective measures for the impact of obesity programmes in changing obesity user behavior. The results indicate that comprehensive multi-component interventions achieve the highest BMI reduction (4.8%) but at significantly higher cost (€380 per student), while single-component approaches offer modest results at lower investment. The complexity-effectiveness-cost gradient demonstrates clear patterns. More specifically, single-component interventions (for example, nutrition only) yield a 1.2% BMI reduction at €45/student, while physical activity alone yields 1.5% at €65/student, meaning that they offer modest, cost-effective entry points but limited impact. Dual-component programs (nutrition + physical activity) yield a 2.3% reduction at €95/student showing synergistic benefits exceeding additive effects. Comprehensive multi-component interventions achieve the highest effectiveness (4.8% BMI reduction) but at substantially increase of the cost (€380/student). These programs integrate nutrition, physical activity, environmental modifications, family engagement, and digital tools. [like the PREVENT EU funded programme (23)]. The sustainability paradox emerges here: while comprehensive programs show highest short-term effectiveness (4.0/5), their resource intensity and coordination complexity may compromise long-term sustainability (3.5/5) compared to triple-component interventions (3.7/5).

### 5.5 Financial constraints

**Figure 7:**
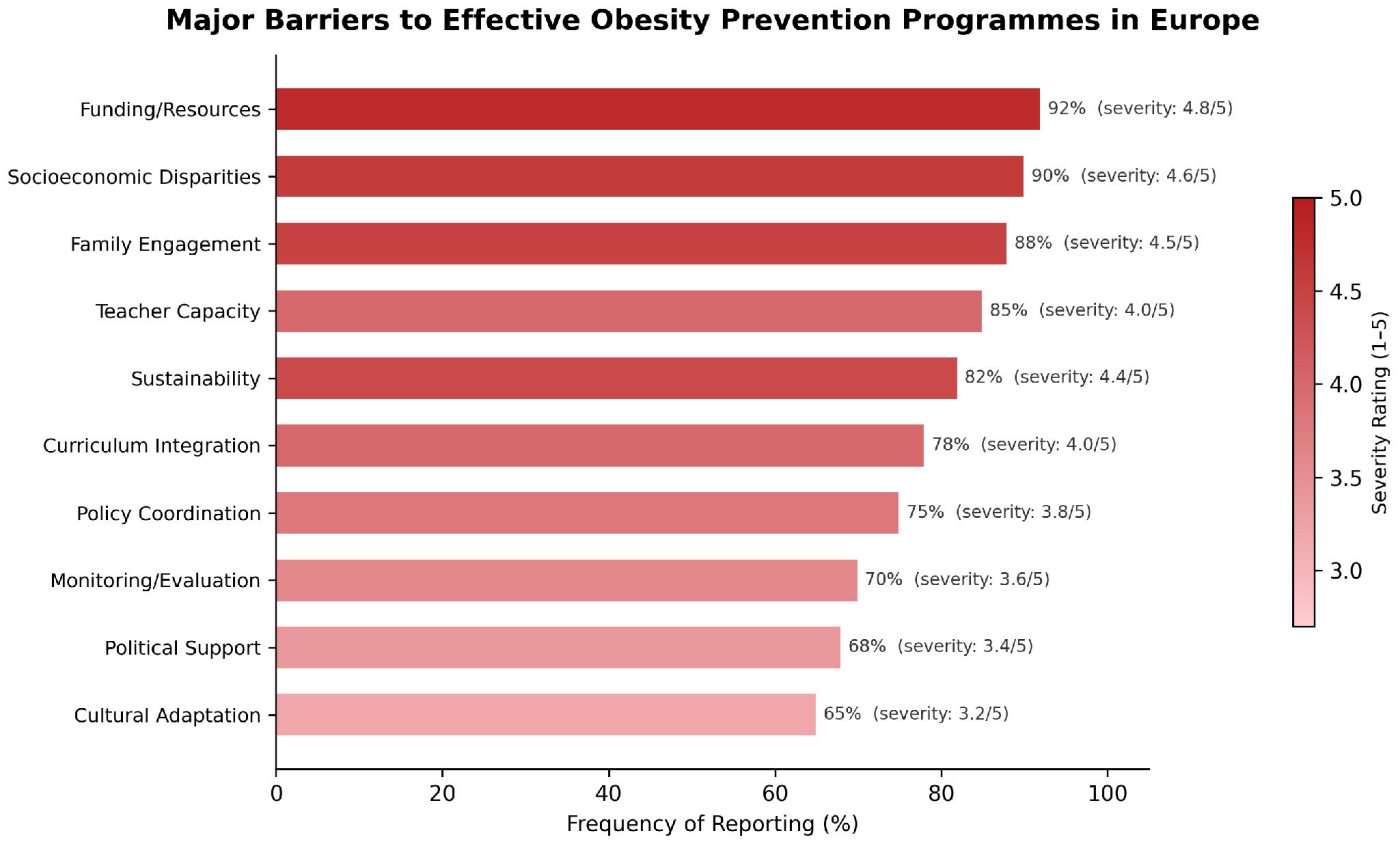
Most frequent barriers toward an effective obesity prevention programmes [sources: Almutairi et al. (75), Taghizadeh et al. (76), and Mahase (77, 78)].

Funding/resources (92%) and socioeconomic disparities (90%) are the most frequently reported barriers, with family engagement and sustainability also presenting significant challenges. Funding and resource constraints represent the most pervasive barrier, reported by 92% of European programs with the highest severity rating (4.8/5). This encompasses insufficient budgets, inadequate equipment, and lack of appropriate facilities across diverse economic contexts. Socioeconomic disparities (90% frequency, 4.6/5 severity) create persistent effectiveness gaps, with low-income families facing compounded barriers including limited access to healthy foods, reduced opportunities for organized physical activities, and competing priorities that make sustained engagement difficult. Family engagement barriers (88% frequency, 4.5/5 severity) stem from parental reluctance due to insufficient knowledge, time constraints from work obligations, and financial limitations. Teacher capacity limitations (85% frequency, 4.0/5 severity) reflect overcrowded curricula prioritizing academic achievement over health promotion, with physical education often receiving lower priority than core subjects. The sustainability challenge (82% frequency, 4.4/5 severity) highlights that while many interventions show positive short-term effects, long-term maintenance remains elusive due to funding cycles, political priorities, and implementation fidelity issues. Figure 7 depicts the most frequent barriers toward an effective obesity prevention programme.

## 6 Conclusions

This review consolidates robust epidemiologic, mechanistic, and implementation evidence establishing childhood and adolescent adiposity as a major, modifiable driver of lifelong cancer risk.

School systems remain indispensable for population reach, but European experience shows that multi-component, school-based programs alone rarely achieve sustained, population-level BMI reductions without structural policies, family engagement, and community and healthcare integration; when present, these layers improve behaviors and intermediate markers yet still face scalability, fidelity, and equity gaps, especially in lower socioeconomic contexts. Fiscal levers such as tiered sugary drink taxes and comprehensive product taxes catalyze consumption shifts and reformulation, but measurable obesity prevalence impacts are limited without parallel actions on physical activity, food environments, marketing, and clinical follow-up, highlighting the need for orchestrated, multisector policy packages with long-term political and financial commitments. Precision prevention is emerging through longitudinal adiposity metrics, metabolic phenotyping, and biomarker-informed risk stratification beyond BMI, enabling earlier, more targeted interventions; however, translation requires interoperable data infrastructures, ethical-by-design analytics, and evaluation frameworks that link behavioral change to cancer-relevant endpoints across decades.

The PREVENT paradigm (23) advances this agenda by integrating co-creation, active behavioral change, digital engagement, trustworthy AI, and poly-parametric assessment within Living Labs that embed schools, families, clinicians, and policy makers, explicitly targeting scalability and legislative pathways for uptake across diverse European contexts. Its Multi-Domain Assessment Matrix and upscaling workstreams directly address historical barriers—implementation fidelity, user acceptability, affordability, and legal constraints—while anchoring outcomes in health, socioeconomic, acceptability, and policy-integration domains that are necessary for durable, system-level change and wider adoption beyond pilots.

Future work should focus on embedding life-course prevention into routine systems through: longitudinal surveillance that captures onset, duration, and cumulative exposure; precision risk models integrating metabolic health, ectopic fat, and social determinants; and sustained school–family–primary care linkages that enable timely referral and reinforced behavior change across settings. Policy packages need to combine universal platforms—such as nutritious school meals meeting strong standards and daily physical activity mandates—with targeted supports for high-risk groups, ensuring equitable benefits and closing persistent socioeconomic gradients in obesity and cancer risk. Digital ecosystems should evolve toward privacy-preserving, explainable decision support that personalize interventions.

## References

[1] Visscher TL Seidell JC. The public health impact of obesity. Annu Rev Public Health. (2001) 22:355–75. doi: 10.1146/annurev.publhealth.22.1.355 11274526

[2] Collaborators GO. Health effects of overweight and obesity in 195 countries over 25 years. New Engl J Med. (2017) 377:13–27. doi: 10.1056/NEJMoa1614362 PMC5477817 28604169

[3] Ahmed SK Mohammed RA. Obesity: Prevalence, causes, consequences, management, preventive strategies and future research directions. Metabol Open. (2025) 27:100375. doi: 10.1016/j.metop.2025.100375 PMC12486175 41041606

[4] Our World in Data. Obesity and Overweight. Based on World Health Organization data (2025). Available online at: https://ourworldindata.org/obesity (Accessed January 15, 2026).

[5] Livingstone B. Epidemiology of childhood obesity in Europe. Eur J Pediatr. (2000) 159:S14–34. doi: 10.1007/PL00014363 11011953

[6] United Nations Regional Information Centre for Western Europe. Europe: One in Three Children Overweight or Obese (2021). Available online at: https://unric.org/en/europe-one-in-three-children-overweight-or-obese/ (Accessed January 15, 2026).

[7] Buoncristiano M Spinelli A Williams J Nardone P Rito AI García-Solano M . Childhood overweight and obesity in Europe: changes from 2007 to 2017. Obes Rev. (2021) 22:e13226. doi: 10.1111/obr.13226 34378305

[8] Centers for Disease Control and Prevention. Obesity and Cancer. (2023). Available online at: https://www.cdc.gov/cancer/obesity/index.htm (Accessed January 15, 2026).

[9] Reinehr T. Obesity in adolescents and cancer risk: causal relationship or epiphenomenon? Lancet Diab Endocrinol. (2020) 8:179–80. doi: 10.1016/S2213-8587(20)30028-0 32027850

[10] Weihe P Spielmann J Kielstein H Henning-Klusmann J Weihrauch-Blüher S. Childhood obesity and cancer risk in adulthood. Curr Obes Rep. (2020) 9:204–12. doi: 10.1007/s13679-020-00387-w 32519271

[11] Flodgren GM Helleve A Lobstein T Rutter H Klepp KI. Primary prevention of overweight and obesity in adolescents: an overview of systematic reviews. Obes Rev. (2020) 21:e13102. doi: 10.1111/obr.13102 32677208

[12] Azevedo LB Stephenson J Ells L Adu-Ntiamoah S DeSmet A Giles EL . The effectiveness of e-health interventions for the treatment of overweight or obesity in children and adolescents: a systematic review and meta-analysis. Obes Rev. (2022) 23:e13373. doi: 10.1111/obr.13373 34747118

[13] Brown T Summerbell C. Systematic review of school-based interventions that focus on changing dietary intake and physical activity levels to prevent childhood obesity: an update to the obesity guidance produced by the National Institute for Health and Clinical Excellence. Obes Rev. (2009) 10:110–41. doi: 10.1111/j.1467-789X.2008.00515.x 18673306

[14] Wolfenden L Wyse R Nichols M Allender S Millar L McElduff P . A systematic review and meta-analysis of whole of community interventions to prevent excessive population weight gain. Prevent Med. (2014) 62:193–200. doi: 10.1016/j.ypmed.2014.01.031 24518005

[15] Vucenik I Stains JP. Obesity and cancer risk: evidence, mechanisms, and recommendations. Ann N Y Acad Sci. (2012) 1271:37–43. doi: 10.1111/j.1749-6632.2012.06750.x PMC3476838 23050962

[16] Ning Y Wang L Giovannucci E. A quantitative analysis of body mass index and colorectal cancer: findings from 56 observational studies. Obes Rev. (2010) 11:19–30. doi: 10.1111/j.1467-789X.2009.00613.x 19538439

[17] Khaodhiar L McCowen KC Blackburn GL. Obesity and its comorbid conditions. Clin Cornerstone. (1999) 2:17–31. doi: 10.1016/S1098-3597(99)90002-9 10696282

[18] Elangovan A Skeans J Landsman M Ali SM Elangovan AG Kaelber DC . Colorectal cancer, age, and obesity-related comorbidities: a large database study. Dig Dis Sci. (2021) 66:3156–63. doi: 10.1007/s10620-020-06602-x 32954457

[19] Yuen MM. Health complications of obesity: 224 obesity-associated comorbidities from a mechanistic perspective. Gastroenterol Clin. (2023) 52:363–80. doi: 10.1016/j.gtc.2023.03.006 37197879

[20] Singhal J Herd C Adab P Pallan M. Effectiveness of school-based interventions to prevent obesity among children aged 4 to 12 years old in middle-income countries: a systematic review and meta-analysis. Obes Rev. (2021) 22:e13105. doi: 10.1111/obr.13105 32725780

[21] Spiga F Tomlinson E Davies AL Moore TH Dawson S Breheny K . Interventions to prevent obesity in children aged 12 to 18 years old. Cochr Datab System Rev. (2024) 5:CD015330. doi: 10.1002/14651858.CD015330.pub2 PMC11102824 38763518

[22] Jebeile H Kelly AS O'Malley G Baur LA. Obesity in children and adolescents: epidemiology, causes, assessment, and management. Lancet Diab Endocrinol. (2022) 10:351–65. doi: 10.1016/S2213-8587(22)00047-X PMC9831747 35248172

[23] PREVENT Consortium. Improving and Upscaling Primary Prevention of Cancer by Addressing Childhood Obesity through Implementation Research: The PREVENT Approach. Horizon Europe Programme. European Union funded research project (2022).

[24] World Health Organization. Cancer Attributable to Obesity. Developed by the International Agency for Research on Cancer. (2012). Available online at: https://gco.iarc.fr/causes/obesity/tools-map (Accessed January 15, 2026).

[25] World Health Organization Regional Office for Europe. Post-COVID Trends in Childhood Obesity: COSI Round 6 (2022-2024). WHO/Europe fact sheet. (2024). Available online at: https://www.who.int/europe/publications/i/item/WHO-EURO-2024-XXXX (Accessed January 15, 2026).

[26] Reilly JJ Kelly J. Long-term impact of overweight and obesity in childhood and adolescence on morbidity and premature mortality in adulthood: systematic review. Int J Obes. (2011) 35:891–8. doi: 10.1038/ijo.2010.222 20975725

[27] Lenz M Richter T Mühlhauser I. The morbidity and mortality associated with overweight and obesity in adulthood: a systematic review. Deutsches Ärzteblatt Int. (2009) 106:641. doi: 10.3238/arztebl.2009.0641 PMC2770228 19890430

[28] Biro FM Wien M. Childhood obesity and adult morbidities. Am J Clin Nutr. (2010) 91:1499S–505S. doi: 10.3945/ajcn.2010.28701B PMC2854915 20335542

[29] Lauby-Secretan B Scoccianti C Loomis D Grosse Y Bianchini F Straif K. Body fatness and cancer–viewpoint of the IARC working group. New Engl J Med. (2016) 375:794–8. doi: 10.1056/NEJMsr1606602 PMC6754861 27557308

[30] IARC Working Group. IARC Handbooks of Cancer Prevention, Volume 16: Body Fatness. International Agency for Research on Cancer. Summary published as Lauby-Secretan B, Scoccianti C, Loomis D, et al. N Engl J Med. 2016; 375(8):794–798. (2016). doi: 10.1056/NEJMsr1606602

[31] Recalde M Pistillo A Davila-Batista V Leitzmann M Romieu I Viallon V . Longitudinal body mass index and cancer risk: a cohort study of 2.6 million Catalan adults. Nat Commun. (2023) 14:3816. doi: 10.1038/s41467-023-39282-y PMC10313757 37391446

[32] Recalde M Pistillo A Viallon V Fontvieille E Duarte-Salles T Freisling H. Body mass index and incident cardiometabolic conditions in relation to obesity-related cancer risk: a population-based cohort study in Catalonia, Spain. Cancer Med. (2023) 12:20188–200. doi: 10.1002/cam4.6603 PMC10587966 37766588

[33] Bibiloni MDM Gallardo-Alfaro L Gómez SF Wärnberg J Osés-Recalde M González-Gross M . Determinants of adherence to the Mediterranean diet in Spanish children and adolescents: the PASOS study. Nutrients. (2022) 14:738. doi: 10.3390/nu14040738 PMC8874357 35215388

[34] Mahamat-Saleh Y Aune D Freisling H Hardikar S Jaafar R Rinaldi S . Association of metabolic obesity phenotypes with risk of overall and site-specific cancers: a systematic review and meta-analysis of cohort studies. Br J Cancer. (2024) 131:1480–95. doi: 10.1038/s41416-024-02857-7 PMC11519895 39317703

[35] Lysaght J Conroy MJ. The multifactorial effect of obesity on the effectiveness and outcomes of cancer therapies. Nat Rev Endocrinol. (2024) 20:701–14. doi: 10.1038/s41574-024-01032-5 39313571

[36] Schmitz SH Aronne LJ. Using second-generation anti-obesity medications. Diab Spectr. (2024) 37:303–12. doi: 10.2337/dsi24-0002 PMC11623031 39649687

[37] Eurostat. Education and Training Statistics Database. (2025). Available online at: https://ec.europa.eu/eurostat/web/education-and-training (Accessed June 3, 2026).

[38] Hawkes C Jewell J Allen K. A food policy package for healthy diets and the prevention of obesity and diet-related non-communicable diseases: the NOURISHING framework. Obes Rev. (2013) 14:159–68. doi: 10.1111/obr.12098 24103073

[39] Oldridge-Turner K Kokkorou M Sing F Klepp KI Rutter H Helleve A . Promoting physical activity policy: the development of the MOVING framework. J Phys Activity Health. (2022) 19:292–315. doi: 10.1123/jpah.2021-0732 35316789

[40] Herstad SH Grewal NK Banik A Klepp KI Knai C Luszczynska A . Adolescents' capacity to take action on obesity: a concurrent controlled before-and-after study of the European CO-CREATE project. Obes Rev. (2023) 24:e13622. doi: 10.1111/obr.13622 37753601

[41] European Commission. EU Action Plan on Childhood Obesity 2014-2020. European Commission Health and Food Safety. (2014). Available online at: https://health.ec.europa.eu/system/files/2016-11/childhoodobesity_actionplan_2014_2020_en_0.pdf

[42] Drouka A Brikou D Causeret C Al Ali Al Malla N Sibalo S Àvila C . Effectiveness of school-based interventions in Europe for promoting healthy lifestyle behaviors in children. Children. (2023) 10:1676. doi: 10.3390/children10101676 PMC10605522 37892339

[43] Porter J Gaskin CJ. Clinical practice guidelines for older adults living with overweight and obesity: a scoping review. Clin Nutr Open Sci. (2024) 56:26–36. doi: 10.1016/j.nutos.2024.04.006

[44] Hercberg S Chat-Yung S Chauliac M. The French national nutrition and health program: 2001-2006-2010. Int J Public Health. (2008) 53:68–77. doi: 10.1007/s00038-008-7016-2 18681335

[45] Weichselbaum E Hooper B Buttriss J Theobald C Sgarabottolo V Combris P . Behaviour change initiatives to promote a healthy diet and physical activity in European countries. Nutr Bull. (2013) 38:85–99. doi: 10.1111/nbu.12011

[46] Gansterer A Moliterno P Neidenbach R Ollerieth C Czernin S Scharhag J . Effect of a web-based nutritional and physical activity intervention with email support (the EDDY Program) on primary school children's BMI Z-score during the COVID-19 pandemic: intervention study. JMIR Pediatr Parent. (2024) 7:e50289. doi: 10.2196/50289 PMC11426922 39298741

[47] WHO Regional Office for Europe. WHO European Regional Obesity Report 2022. Copenhagen: World Health Organization Regional Office for Europe (2022). Available online at: https://www.who.int/europe/publications/i/item/9789289057738

[48] Gorasso V De Smedt D Vandevijvere S De Clercq E Van der Heyden J Doggen K . Impact of overweight on the burden of non-communicable diseases in Belgium: the WaIST project. Eur J Public Health. (2020) 30:ckaa166-412. doi: 10.1093/eurpub/ckaa166.412

[49] Ryom K Bloch P Toft U Høeg D Thomsen LT Allender S . Involving national stakeholders in a systems approach to map causes and proposed actions to prevent childhood obesity in Denmark: the generation healthy kids project. BMC Public Health. (2024) 24:1–13. doi: 10.21203/rs.3.rs-3879696/v1

[50] Thomsen LT Schmidt-Persson J Damsgaard CT Krustrup P Grøntved A Krølner RF . Generation Healthy Kids: protocol for a cluster-randomized controlled trial of a multi-component and multi-setting intervention to promote healthy weight and wellbeing in 6-11-year-old children in Denmark. PLoS ONE. (2024) 19:e0308142. doi: 10.1371/journal.pone.0308142 PMC11620443 39636875

[51] Ministére du Travail de la Santé des Solidarités et des Familles. Programme National Nutrition Santé 2026-2030 (PNNS 5) (2026). French Ministry of Health, 72 pp. Available online at: https://sante.gouv.fr/IMG/pdf/pnns_5_2026-2030.pdf (Accessed June 25, 2026).

[52] Sharma M. International school-based interventions for preventing obesity in children. Obes Rev. (2007) 8:155–67. doi: 10.1111/j.1467-789X.2006.00268.x 17300280

[53] Müller M Asbeck I Mast M Langnäse K Grund A. Prevention of obesity—more than an intention. Concept and first results of the Kiel Obesity Prevention Study (KOPS). Int J Obes. (2001) 25:S66–S74. doi: 10.1038/sj.ijo.0801703 11466593

[54] Mannion E Bihrmann K Plachta-Danielzik S Müller MJ Bosy-Westphal A Ritz C. Exploring the effect of an obesity-prevention intervention on various child subgroups: a post hoc subgroup analysis of the Kiel obesity prevention study. Nutrients. (2024) 16:3220. doi: 10.3390/nu16183220 PMC11434954 39339820

[55] Angelopoulos P Milionis H Grammatikaki E Moschonis G Manios Y. Changes in BMI and blood pressure after a school based intervention: the CHILDREN study. Eur J Public Health. (2009) 19:319–25. doi: 10.1093/eurpub/ckp004 19208697

[56] Berezvai Z Vitrai J Tóth G Brys Z Bakacs M Joó T. Long-term impact of unhealthy food tax on consumption and the drivers behind: a longitudinal study in Hungary. Health Policy. (2024) 146:105098. doi: 10.1016/j.healthpol.2024.105098 38851004

[57] Rurik I Torzsa P Szidor J Móczár C Iski G Albók É . A public health threat in Hungary: obesity, 2013. BMC Public Health. (2014) 14:798. doi: 10.1186/1471-2458-14-798 PMC4143555 25096526

[58] Spinelli A Lamberti A Nardone P Andreozzi S Galeone D editors. Sistema di sorveglianza OKkio alla SALUTE: risultati 2010 [The Surveillance System OKkio alla SALUTE: Results 2010]. Rome: Istituto Superiore di Sanit0. (Rapporti ISTISAN 12/14). Italian (2012). p. xii, 139.

[59] Spinelli A Nardone P Buoncristiano M Salvatore MA Bucciarelli M Andreozzi S . Sovrappeso e obesità nei bambini: il contributo del Programma nazionale Guadagnare Salute [Childhood overweight and obesity: the contribution made by the national pro gramme Guadagnare Salute]. Boll Epidemiol Naz. (2021) 2:39–45 (Italian). doi: 10.53225/BEN_035

[60] Kobes A Kretschmer T Timmerman MC. Prevalence of overweight among Dutch primary school children living in JOGG and non-JOGG areas. PLoS ONE. (2021) 16:e0261406. doi: 10.1371/journal.pone.0261406 PMC8682899 34919583

[61] Goiana-da Silva F Cruz-e Silva D Gregório MJ Miraldo M Darzi A Araújo F. The future of the sweetened beverages tax in Portugal. Lancet Public Health. (2018) 3:e562. doi: 10.1016/S2468-2667(18)30240-8 30522681

[62] Goiana-da Silva F Severo M Cruz e Silva D Gregório MJ Allen LN Muc M . Projected impact of the Portuguese sugar-sweetened beverage tax on obesity incidence across different age groups: a modelling study. PLoS Med. (2020) 17:e1003036. doi: 10.1371/journal.pmed.1003036 PMC7067376 32163412

[63] Giralt M Albaladejo R Tarro L Moriña D Arija V Solá R. A primary-school-based study to reduce prevalence of childhood obesity in Catalunya (Spain)-EDAL-Educació en alimentació: study protocol for a randomised controlled trial. Trials. (2011) 12:54. doi: 10.1186/1745-6215-12-54 PMC3052179 21352597

[64] Tarro L Llauradó E Albaladejo R Mori na D Arija V Solá R . A primary-school-based study to reduce the prevalence of childhood obesity–the EdAl (Educació en Alimentació) study: a randomized controlled trial. Trials. (2014) 15:58. doi: 10.1186/1745-6215-15-58 PMC3926975 24529258

[65] Tarro L. Llauradó E, Moriña D, Solá R, Giralt M. Follow-up of a healthy lifestyle education program (the Educació en Alimentació Study): 2 years after cessation of intervention. J Adolesc Health. (2014) 55:782–9. doi: 10.1016/j.jadohealth.2014.06.020 25193385

[66] Nyberg G Sundblom E. Norman Å, Bohman B, Hagberg J, Schäfer Elinder L. Effectiveness of a universal parental support programme to promote healthy dietary habits and physical activity and to prevent overweight and obesity in 6-year-old children: the Healthy School Start Study, a cluster-randomised controlled trial. PLoS ONE. (2015) 10:e0116876. doi: 10.1371/journal.pone.0116876 PMC4332680 25680096

[67] Nyberg G. Norman Å, Sundblom E, Zeebari Z, Schäfer Elinder L. Effectiveness of a universal parental support programme to promote health behaviours and prevent overweight and obesity in 6-year-old children in disadvantaged areas, the Healthy School Start Study II, a cluster-randomised controlled trial. Int J Behav Nutr Phys Activ. (2016) 13:4. doi: 10.1186/s12966-016-0327-4 PMC4721005 26795378

[68] Norman Å Zeebari Z Nyberg G Schäfer Elinder L. Parental support in promoting children's health behaviours and preventing overweight and obesity–a long-term follow-up of the cluster-randomised Healthy School Start Study II trial. BMC Pediatr. (2019) 19:104. doi: 10.1186/s12887-019-1467-x PMC6458763 30975106

[69] Rogers NT Pell D Mytton OT Penney TL Briggs A Cummins S . Changes in soft drinks purchased by British households associated with the UK soft drinks industry levy: a controlled interrupted time series analysis. BMJ Open. (2023) 13:e077059. doi: 10.1136/bmjopen-2023-077059 PMC10711915 38052470

[70] Scarborough P Adhikari V Harrington RA Elhussein A Briggs A Rayner M . Impact of the announcement and implementation of the UK Soft Drinks Industry Levy on sugar content, price, product size and number of available soft drinks in the UK, 2015-19: a controlled interrupted time series analysis. PLoS Med. (2020) 17:e1003025. doi: 10.1371/journal.pmed.1003025 PMC7012398 32045418

[71] Ruiz-Hermosa A Sánchez-López M Castro-Piñero J Grao-Cruces A Camiletti-Moirón D Martins J . The Erasmus+ EUMOVE project-a school-based promotion of healthy lifestyles to prevent obesity in European children and adolescents. Eur J Public Health. (2024) 34:95561. doi: 10.1093/eurpub/ckae113 PMC11430908 39074353

[72] De Craemer M Cardon G Decraene M Androutsos O Moreno L Iotova V . Longitudinal changes in preschoolers' adiposity indicators according to compliance with 24-hour movement behavior guidelines: results from the ToyBox-study. BMC Public Health. (2024) 24:3115. doi: 10.1186/s12889-024-20626-2 PMC11555805 39528995

[73] Buck C Tkaczick T Pitsiladis Y De Bourdehaudhuij I Reisch L Ahrens W . Objective measures of the built environment and physical activity in children: from walkability to moveability. J Urban Health. (2015) 92:24–38. doi: 10.1007/s11524-014-9915-2 PMC4338118 25380722

[74] Porri D Wasniewska M Luppino G Morabito LA La Rosa E Pepe G . The rising burden of childhood obesity: prevention should start in primary school. Nutrients. (2025) 17:650. doi: 10.3390/nu17040650 PMC11858576 40004978

[75] Almutairi N Burns S Portsmouth L. Barriers and enablers to the implementation of school-based obesity prevention strategies in Jeddah, KSA. Int J Qual Stud Health Well-being. (2022) 17:2135197. doi: 10.1080/17482631.2022.2135197 PMC9590444 36263729

[76] Taghizadeh S Alesaeidi S Jafari-Koshki T Valizadeh-Otaghsara SM Poursheikhali A Tousi AZ . Trend Impact Analysis (TIA) of community-based futures study for pediatric obesity in Iran. BMC Pediatr. (2023) 23:66. doi: 10.1186/s12887-023-03880-y PMC9905010 36750801

[77] Mahase E. Obesity: Only half of England has access to comprehensive weight loss services. BMJ. (2024) 386:q1950. doi: 10.1136/bmj.q1950 39260875

[78] Mahase E. Obesity: Government accused of kicking problem “into the long grass” despite growing crisis. BMJ. (2025) 388:r229. doi: 10.1136/bmj.r229 39890126

[79] Nittari G Scuri S Petrelli F Pirillo I di Luca NM Grappasonni I. Fighting obesity in children from European World Health Organization member states. Epidemiological data, medical-social aspects, and prevention programs. Clin Ter. (2019) 170:e22330. doi: 10.7417/CT.2019.2137 31173054

[80] Saleh M Ba-Break M Abahussin A. Barriers and facilitators of school-based obesity prevention interventions: a qualitative study from the perspectives of primary school headteachers. J Health, Popul Nutr. (2024) 43:217. doi: 10.1186/s41043-024-00713-1 PMC11658294 39696660

[81] Regber S. Barriers and facilitators of health promotion and obesity prevention in early childhood: a focus on parents. Results from the IDEFICS Study. University of Gothenburg. Doctoral dissertation (2014).

